# Correlations of expression of nuclear and mitochondrial genes in triploid fish

**DOI:** 10.1093/g3journal/jkac197

**Published:** 2022-08-04

**Authors:** Jialin Cui, Hong Zhang, Xin Gao, Xueyin Zhang, Mengxue Luo, Li Ren, Shaojun Liu

**Affiliations:** State Key Laboratory of Developmental Biology of Freshwater Fish, College of Life Sciences, Hunan Normal University, Changsha 410081, Hunan, P.R. China; Guangdong Laboratory for Lingnan Modern Agriculture, South China Agricultural University, Guangzhou 510642, Guangdong, P.R. China; State Key Laboratory of Developmental Biology of Freshwater Fish, College of Life Sciences, Hunan Normal University, Changsha 410081, Hunan, P.R. China; Guangdong Laboratory for Lingnan Modern Agriculture, South China Agricultural University, Guangzhou 510642, Guangdong, P.R. China; State Key Laboratory of Developmental Biology of Freshwater Fish, College of Life Sciences, Hunan Normal University, Changsha 410081, Hunan, P.R. China; Guangdong Laboratory for Lingnan Modern Agriculture, South China Agricultural University, Guangzhou 510642, Guangdong, P.R. China; State Key Laboratory of Developmental Biology of Freshwater Fish, College of Life Sciences, Hunan Normal University, Changsha 410081, Hunan, P.R. China; Guangdong Laboratory for Lingnan Modern Agriculture, South China Agricultural University, Guangzhou 510642, Guangdong, P.R. China; State Key Laboratory of Developmental Biology of Freshwater Fish, College of Life Sciences, Hunan Normal University, Changsha 410081, Hunan, P.R. China; Guangdong Laboratory for Lingnan Modern Agriculture, South China Agricultural University, Guangzhou 510642, Guangdong, P.R. China; State Key Laboratory of Developmental Biology of Freshwater Fish, College of Life Sciences, Hunan Normal University, Changsha 410081, Hunan, P.R. China; Guangdong Laboratory for Lingnan Modern Agriculture, South China Agricultural University, Guangzhou 510642, Guangdong, P.R. China; State Key Laboratory of Developmental Biology of Freshwater Fish, College of Life Sciences, Hunan Normal University, Changsha 410081, Hunan, P.R. China; Guangdong Laboratory for Lingnan Modern Agriculture, South China Agricultural University, Guangzhou 510642, Guangdong, P.R. China

**Keywords:** mitochondrial-nuclear correlation, gene expression, embryonic development, triploid fish

## Abstract

The expression of nuclear and mitochondrial genes, as well as their coordinated control, regulates cell proliferation, individual development, and disease in animals. However, the potential coregulation between nuclear and mitochondrial genes is unclear in triploid fishes. The two triploids (R_2_C and RC_2_) with distinct mitochondrial genomes but similar nuclear genomes exhibit different embryonic development times and growth rates. They are an excellent model for studying how nuclear and mitochondrial genes coordinate. Here, we performed the mRNA-seq of four stages of embryonic development (blastula, gastrula, segmentation, and hatching periods) in the two triploids (R_2_C and RC_2_) and their diploid inbred parents (red crucian carp and common carp). After establishing the four patterns of mitochondrial and nuclear gene expression, 270 nuclear genes regulated by mitochondrial genes were predicted. The expression levels of *APC16* and *Trim33* were higher in RC_2_ than in R_2_C, suggesting their potential effects on regulating embryonic development time. In addition, 308 differentially expressed genes filtered from the list of nuclear-encoded mitochondrial genes described by Mercer *et al.* in 2011 were considered potential genes for which nuclear genes regulate mitochondrial function. The findings might aid in our understanding of the correlation between mitochondrial and nuclear genomes as well as their synergistic effects on embryonic development.

## Introduction

Mitochondria are intracellular energy-producing units that provide energy for a series of cellular activities, including Na^+^/K^+^ ATPase pump activity, endocytosis, protein synthesis, and some other processes (Brown 2008; Tao *et al.* 2014). The capacity of mitochondria to undergo reprogramming between different situations is important for all cell types (Schirrmacher 2020). Consequently, normal levels of mitochondrial (MT) fission and fusion are important for proper cellular function and development, while abnormal variations cause disease phenotypes in animals (Chan 2006; Luo *et al.* 2018). The MT genome in vertebrates is compact, generally spanning 16–17 kb in size, with overlapping coding sequences on the heavy and light strands for several genes and a complete lack of introns (Barrell *et al.* 1979; Satoh *et al.* 2016).

To adapt to the environmental challenges, nuclear-mitochondrial genetic interactions and collaboration occur continuously (Fetterman and Ballinger 2019). Endosymbiosis and the coevolution of the mitochondrial (MT) and nuclear (NU) genomes over 1.5 billion years resulted in eukaryotes (Dunham-Snary *et al.* 2018). The dynamic nature of the MT genome probably needs to be coordinated by the rapid coevolution of the MT proteins encoded in the NU genome. It is likely that interactions between MT and NU genes play an important role during speciation, e.g. in yeast (Lee *et al.* 2008). Previous experiments showed that pairing MT and NU genomes from two different strains resulted in reduced fitness (Luo *et al.* 2013). Another study demonstrated that the nuclear-mitochondrial genome combination significantly altered metabolic efficiency and body composition. The influence of MT DNA on regulating NU gene expression was clearly demonstrated by comparative gene expression analysis in adipose tissues (Dunham-Snary *et al.* 2018).

Growth heterosis in hybrid fish could benefit aquaculture (Houston *et al.* 2020). Two triploid fishes (R_2_C and RC_2_) were obtained from back-crossing of the male allotetraploid (4n = 200, 4nAT) of red crucian carp (*Carassius auratus* red var., RCC) × common carp (*Cyprinus carpio* L., CC) with female RCC and CC, respectively (Shen *et al.* 2006; Chen *et al.* 2009). The types of MT genomes in R_2_C and RC_2_ are the same as in RCC and CC, respectively (Ren *et al.* 2021). The combined effects of dosage compensation and incomplete dominance were predicted by gene expression profiling and had potential effects on their growth heterosis (Ren *et al.* 2019). The different expressions of GH/IGF axis genes were detected in the triploid fish and considered as one of the factors for growth heterosis (Zhong *et al.* 2012). This study focused on the expression profiles of NU and MT genes during embryonic development. The different expression patterns of NU and MT genes in R_2_C and RC_2_, which possess distinct MT genomes but similar NU genomes, would provide insights into the mitochondrial-nuclear correlations in triploid fishes.

## Materials and methods

### Ethics statement

In this study, all experiments were approved by the Animal Care Committee of Hunan Normal University and followed the stated guidelines of the Administration of Affairs Concerning Animal Experimentation of China and the ARRIVE. Fish crossing and embryo collecting were approved by the Animal Care Committee and Protection Station of Polyploidy Fish of Hunan Normal University (approval ID: 04/2018).

### Experimental materials

**Fig. 1.**
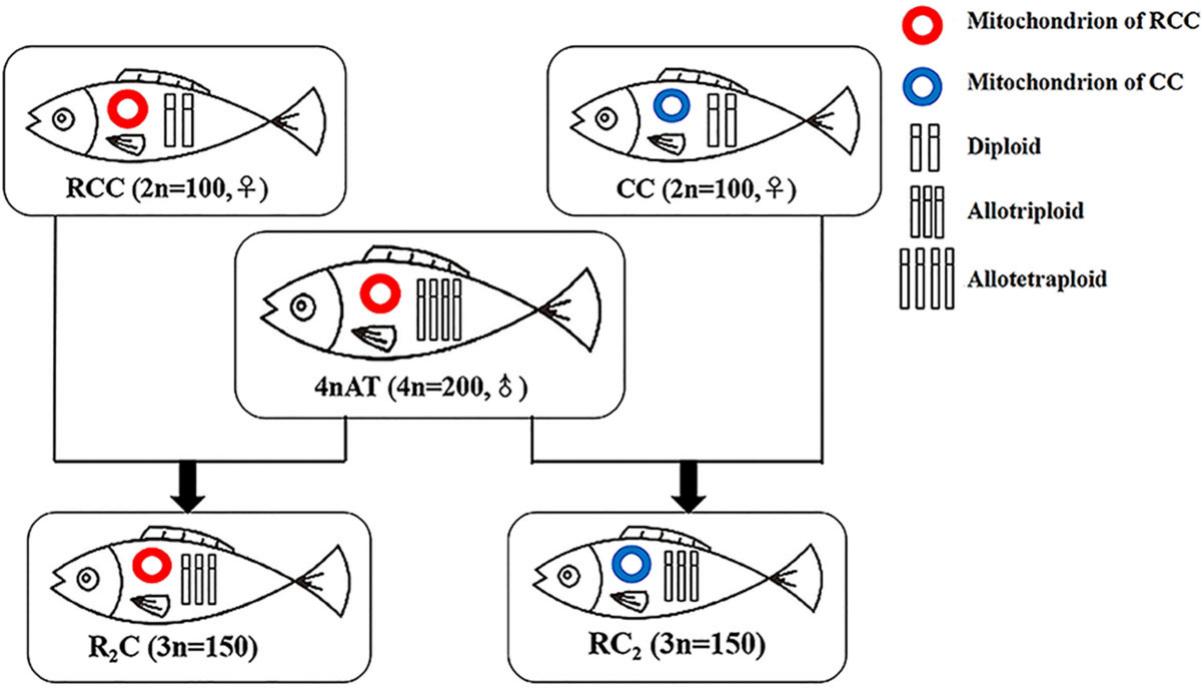
Two allotriploids obtaining from interploidy crossing of the allotetraploid fish with the two diploid parents.

Diploid red crucian carp (*C. auratus* red var., RCC), diploid common carp (*C. carpio* L., CC), and allotetraploid fish (4nAT) derived from RCC (♀) × CC (♂) were obtained from the Engineering Center of Polyploidy Fish Breeding of the National Education Ministry (Hunan Normal University, Hunan, China; Liu *et al.* 2016). During the breeding season (from April to June), experimental self-crossings of RCC and CC were performed. Simultaneously, the two intercrossings were carried out (Fig. 1). In one cross (R_2_C), RCC and 4nAT were used as the maternal and paternal parents, respectively. In the other cross (RC_2_), the maternal parent was changed to CC. R_2_C carried two sets of RCC chromosomes, one set of CC chromosomes and the RCC MT genome, while RC_2_ carried two sets of CC chromosomes, one set of RCC chromosomes and the CC MT genome (Ren *et al.* 2019). All embryos of the four types of fish were incubated with flowing water at 17–18°C. Culture dishes of R_2_C and RC_2_ were selected at random to continuously observe the embryonic development and record the corresponding time (Tsai *et al.* 2013). We collected embryos from all four crosses during the four stages of embryonic development, including blastula (Oblong), gastrula (50%-Epiboly), segmentation (3-Somite), and hatching periods (1 h after hatching; Kimmel *et al.* 1995). To exclude the effects of environmental contamination, all embryo samples were repeatedly rinsed with DEPC-treated water (Sangon Biotech Co., Ltd., Shanghai, China).

### mRNA-seq sequencing

All samples used for mRNA sequencing were stored at −80°C. After DNase treatment, total RNA was extracted from mixed embryos of the same fish at the same developmental stage and then used to construct mRNA-seq libraries according to the manufacturer’s instructions. For each sample, a paired-end (2 × 150 bp) mRNA-seq library was constructed using a NovaSeq 6000 Sequencing System (Illumina, San Diego, CA, USA). The raw sequencing data underwent initial quality control utilizing FastQC. Then, the low-quality bases and adapters were removed using Trimmomatic (Bolger *et al.* 2014).

### Expression of mitochondrial genes in embryonic transcriptomes

The mRNA-seq reads collected from RCC, CC, and the two triploids (R_2_C and RC_2_) at four embryonic development stages were filtered and then mapped to 13 MT protein-coding genes (*ND1*, *ND2*, *COX I*, *COX II*, *ATP8*, *ATP6*, *COX III*, *ND3*, *ND4L*, *ND4*, *ND5*, *ND6*, and *CYTB*) using Salmon software (Patro *et al.* 2017) with default options. The annotations of the MT genomes were obtained from their respective annotated sequence files (NCBI accession Nos. AY714387.1 and KF856965.1). The number of mapped reads and transcripts per million values were obtained from the output results. Paired-samples *t*-test analyses were used to assess the differences in MT gene expression in the four comparisons (Comparison 1: RCC *vs.* R_2_C, Comparison 2: R_2_C *vs.* RC_2_, Comparison 3: CC *vs.* RC_2_, and Comparison 4: RCC *vs.* CC).

### Expression of nuclear genes in embryonic transcriptomes

The mRNA-seq reads of RCC, CC, and two triploids were mapped to the predicted genome coding sequences in RCC (Genome Warehouse in BIG Data Center BioProject No. PRJCA001234) or CC (NCBI accession No. PRJNA510861), respectively (Xu *et al.* 2019; Luo *et al.* 2020). These read-mapped analyses were conducted using Salmon software with default options (Patro *et al.* 2017). Gene annotations were performed with BLASTX searches in NCBI, Gene Ontology (GO), and Swiss-Prot databases. The gene expression values of RCC and CC were assessed based on the mapped reads of their respective transcripts, while the gene expression values of the two triploids were assessed based on the average values of mapped reads of the two reference genomes in each gene. Differential expression (DE) analysis was performed using the DESeq2 package based on the threshold of |log2-fold change| >1.2, *P*-value < 0.01 and a 1% false discovery rate (<0.01) in three biological replicates.

### Interaction patterns of mitochondrial and nuclear genes

The expression levels of NU transcriptomes in R_2_C and RC_2_ of four stages of embryonic development were investigated to screen for differentially expressed genes (DEGs). GO and kyoto encyclopedia of genes and genomes (KEGG) analyses were performed on the DEGs. In comparison with the two triploids, the four patterns of NU and MT gene expression were classified as below: (1) up-regulated MT genes and up-regulated NU genes in R_2_C; (2) up-regulated MT genes and down-regulated NU genes in R_2_C; (3) down-regulated MT genes and up-regulated NU genes in R_2_C; (4) down-regulated MT genes and down-regulated NU genes in R_2_C.

## Results

### Embryonic development time between two triploids

The embryonic development of the two allotriploids (R_2_C and RC_2_) was observed from the zygote to the hatching period. Fifty-five minutes after fertilization, the embryos of R_2_C begin to cleave, while the embryos of RC_2_ need 60 min. Similarly, at 75 h 45 min after fertilization, the embryos of R_2_C begin to hatch, while the embryos of RC_2_ begin to hatch at 76 h 12 min after fertilization. We observed that each stage of embryonic development took more time in RC_2_ than in R_2_C (Table 1).

**Table 1.** Comparison of embryonic development between R_2_C and RC_2_.

	R_2_C	RC_2_
Fertilization	0 min	0 min
Cleavage period (1-cell)	55 min	60 min
Cleavage period (2-cell)	1 h 20 min	1 h 25 min
Blastula period (256-cell)	3 h 30 min	3 h 43 min
Blastula period (Oblong)	5 h 26 min	5 h 52 min
Blastula period (Done)	6 h 11 min	6 h 26 min
Gastrula period (50%-Epiboly)	8 h 5 min	8 h 18 min
Segmentation period (3-Somite)	14 h 28 min	14 h 53 min
Hatching period	75 h 45 min	76 h 12 min

Water temperature: 17–18°C.

### Differential expression of mitochondrial genes among diploids and triploids

After sequencing, 429.38 Gb raw data were obtained from 48 transcriptomes, and 1.39 billion clean reads (417.24 Gb) were retained for further analysis after quality checking (File S1: Table S1). The mRNA-seq reads of the two triploids were mapped to the combined MT genomes of RCC (NCBI accession No. AY714387.1) and CC (NCBI accession No. KF856965.1). The 99.8% reads of R_2_C were mapped to the MT genome of its maternal RCC, while the 99.83% ones of RC_2_ were mapped to the MT genome of its maternal CC (File S1: Table S2). These results showed maternal inheritance in the two triploids.

**Fig. 2.**
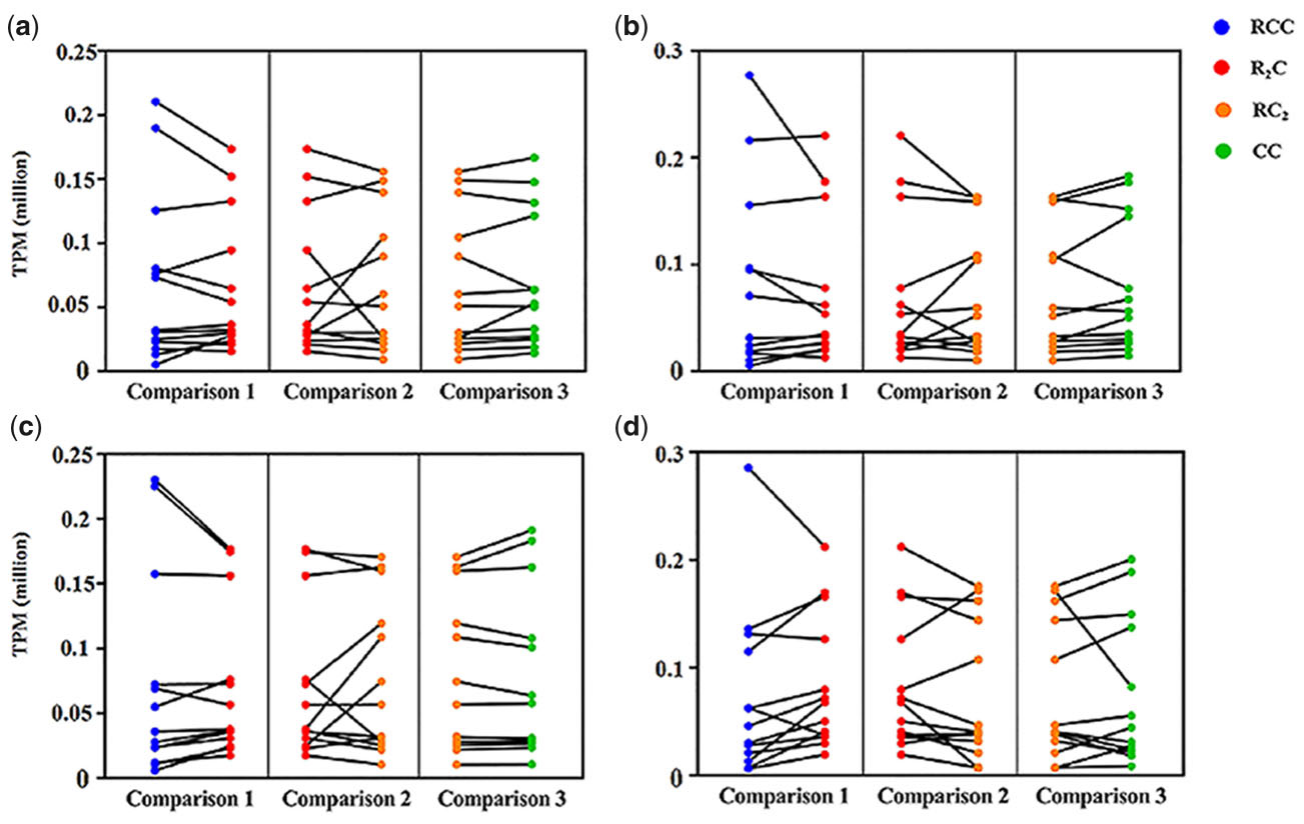
Comparison of expression levels for MT genes between the two triploids and their maternal parents. a) Blastula period; (b) gastrula period; (c) segmentation period; and (d) hatching period.

DE analyses of 13 MT protein-coding genes between the triploids and their corresponding maternal parents were used to detect MT gene expression after hybridization. No significant DE in these 13 genes showed the high conservatism of MT inheritance (Fig. 2). However, the *P*-values of DE in Comparisons 1 and 3 could reflect different degrees of slight changes in MT gene expression (Table 2). The lowest value in RCC *vs.* R_2_C (Comparisons 1) was detected in the hatching period (*P = *0.09), while the lowest value in CC *vs.* RC_2_ (Comparisons 3) was found in the gastrula period. These results suggest diverse changes in MT gene expression during embryonic development.

**Table 2.** Distribution of *P*-value obtained from the paired *t*-test analyses.

		Blastula	Gastrula	Segmentation	Hatching
Comparison 1	RCC *vs.* R_2_C	0.8926	0.9999	0.5417	0.0942
Comparison 2	R_2_C *vs.* RC_2_	0.9460	0.9999	0.9999	0.2439
Comparison 3	CC *vs.* RC_2_	0.1465	0.1272	0.6848	0.4973

DE analyses of 13 MT protein-coding genes between the two triploids will help us understand the differences in RCC and CC MT gene expression in the triploids (Comparison 2). The lowest value was detected in the hatching period (*P = *0.2439), reflecting that the most expression changes of the four stages of embryonic development occurred in the hatching period (Table 2).

### Changes in mitochondrial gene expression accompanied by embryonic development

Expression trends across the four embryonic development stages were observed in each MT protein-coding gene. The gradually increased expression trend accompanied by embryonic development was found in most MT genes, including *ND1*, *ND2*, *COX I*, *COX II*, *ATP8*, *ATP6*, *COX III*, *ND3*, *ND4*, *ND5*, *ND6*, and *CYTB* (File S1: Fig. S1). The expression trends of the 5 MT genes (*COX I*, *ATP8*, *COX III*, *ND5*, and *CYTB*) during embryonic development were different between R_2_C and RCC, and 5 genes (*ND2*, *ATP6*, *ND4L*, *ND4*, and *ND6*) exhibited different trends in expression between RC_2_ and CC (File S1: Fig. S1, Tables S3 and S4).

### Differential expression of nuclear genes among diploids and triploids

**Fig. 3.**
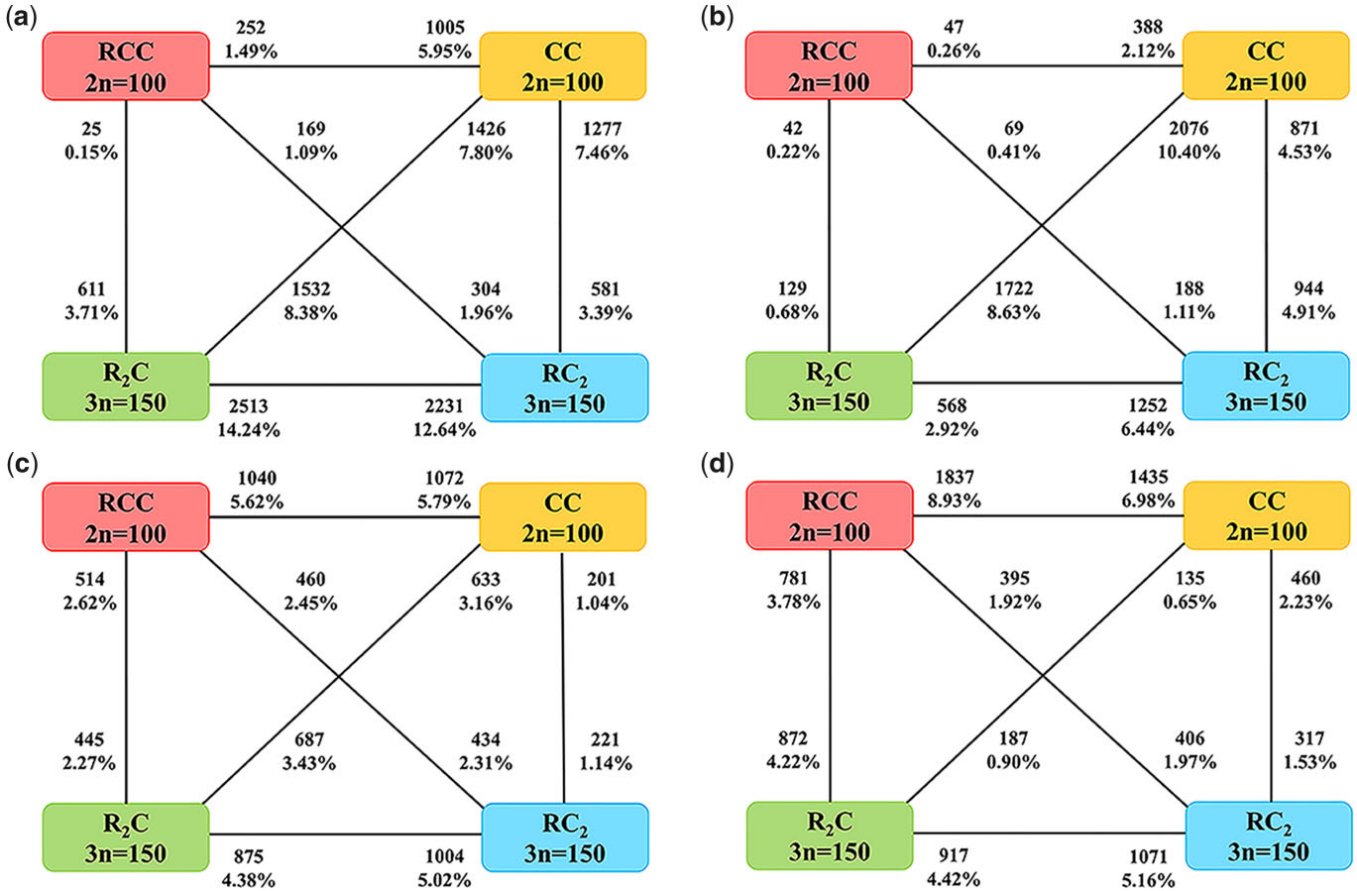
Nuclear DEGs for the four stages of embryonic development among diploids and triploids. a) Blastula period; (b) gastrula period; (c) segmentation period; and (d) hatching period.

We investigated the global expression levels of NU transcriptomes among diploids and triploids and obtained DEGs for the four stages of embryonic development (Fig. 3). The proportions of DEGs between R_2_C and RC_2_ in the blastula period was 26.88% (up-regulated in R_2_C: 2,513, up-regulated in RC_2_: 2,231); in the gastrula period was 9.36% (up-regulated in R_2_C: 568, up-regulated in RC_2_: 1,252); in the segmentation period was 9.40% (up-regulated in R_2_C: 875, up-regulated in RC_2_: 1,004); in the hatching period was 9.58% (up-regulated in R_2_C: 917, up-regulated in RC_2_: 1,071).

### Mitochondrial genes regulating nuclear gene expression

**Fig. 4.**
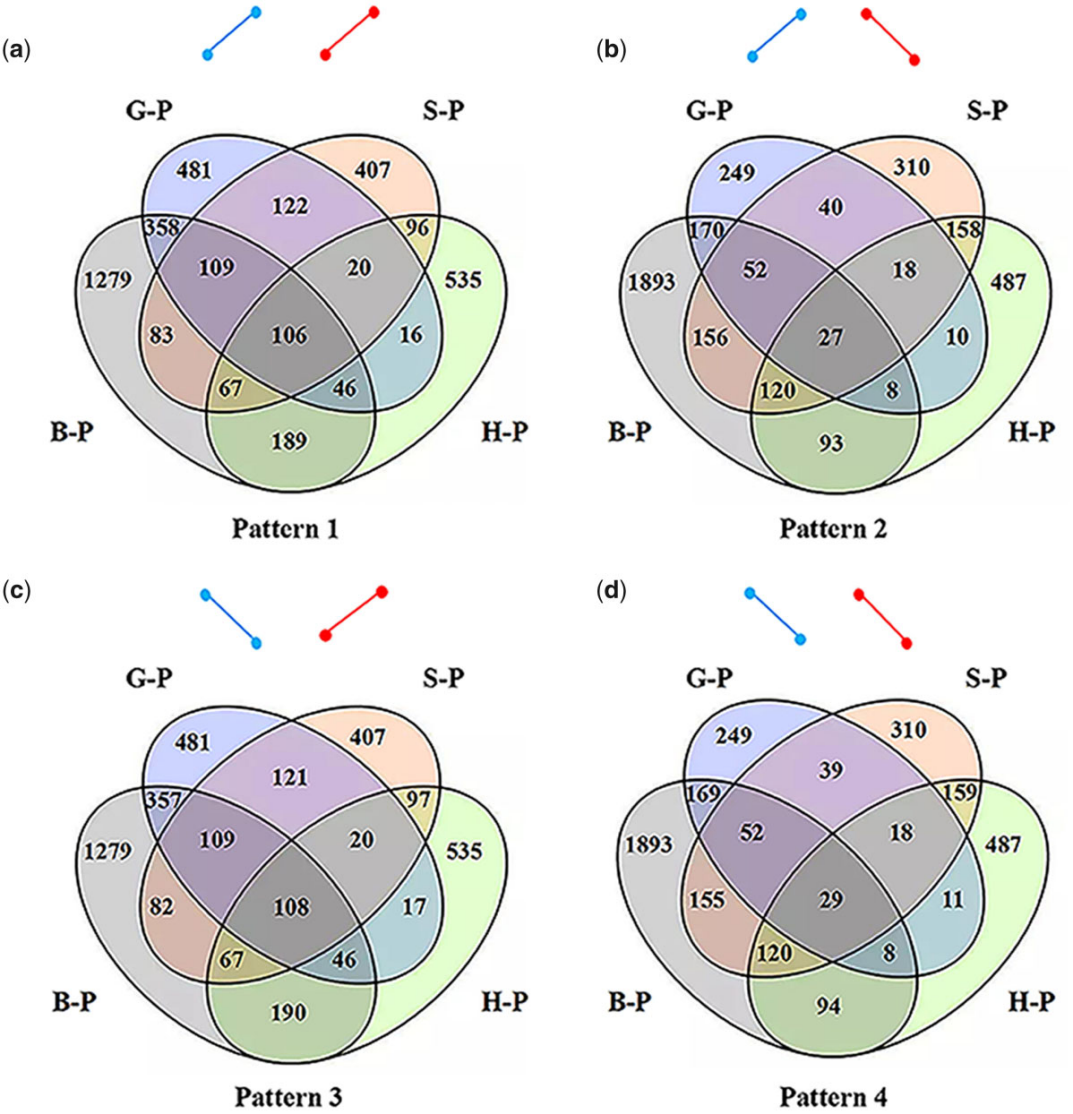
Four patterns of expression trends for MT and NU genes. a) Pattern 1 represents the same expression pattern (up-regulation in MT genes of RC_2_ and up-regulation in NU genes of RC_2_) in the four development stages. b–d) represent patterns 2–4, respectively. Blue line represents change trends of MT gene expression from R_2_C and RC_2_. Red represents change trends of NU gene expression from R_2_C and RC_2_. B-P, blastula period; G-P, gastrula period; S-P, segmentation period; H-P, hatching period.

**Fig. 5.**
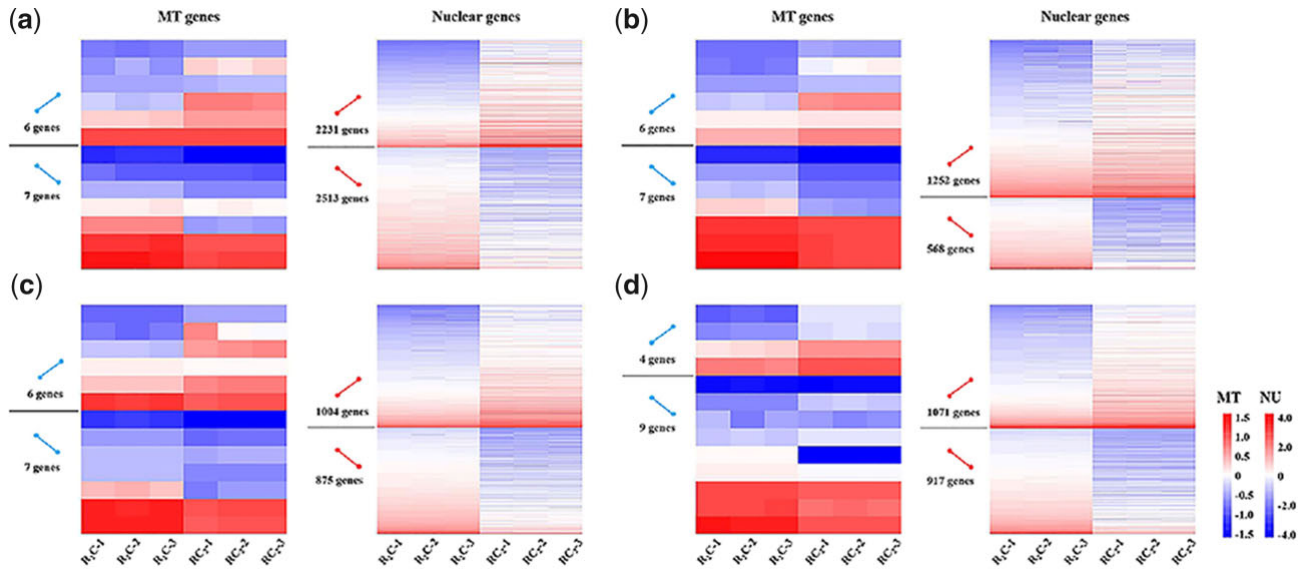
Expression trends of MT and NU gene during embryonic development. a) Blastula period; (b) gastrula period; (c) segmentation period; and (d) hatching period. Blue line represents change trends of MT gene expression from R_2_C and RC_2_. Red line represents change trends of NU gene expression from R_2_C and RC_2_.

Compared with the two triploids, the same or opposite directions of both MT and NU gene expression trends across the four embryonic development stages give us an opportunity to investigate how MT gene expression regulates NU gene expression. MT gene expression positively regulated NU gene expression across the four development stages (patterns 1 and 4 in Fig. 4), while MT gene expression negatively regulated NU gene expression (patterns 2 and 3 in Fig. 4). For the 13 MT genes, the expression of 6, 6, 6, and 4 genes was increased in RC_2_ compared to R_2_C. The expression of 2,231 (47.03%), 1,252 (68.79%), 1,004 (53.43%), and 1,071 (53.87%) NU genes was increased in RC_2_ than in R_2_C (Fig. 5). The 106 genes in pattern 1, 27 genes in pattern 2, 108 genes in pattern 3, and 29 genes in pattern 4 exhibited stable patterns of MT and NU gene expression across the four development stages, reflecting the potential regulation from MT to NU genes (Fig. 4). Interestingly, the shared genes were clearly more in RC_2_ (sum of patterns 1 and 3: 214 genes) than in R_2_C (sum of patterns 2 and 4: 56 genes) under this potential regulation (Fig. 4). This result suggested that MT genes from CC species were more likely to result in increased NU gene expression in RC_2_ than those from R_2_C. GO analysis exhibited that the cell cycle-related genes (*Trim33 and APC16*) were up-regulated in RC_2_ than in R_2_C (File S1: Fig. S2).

### Nuclear genes regulating expression of mitochondrial genes

MT activity is regulated by a series of NU genes. Although no significant DE of MT genes was detected among the diploids and triploids, we focused on potential 417 nuclear-encoded mitochondrial genes (NEMGs) in the transcriptome data, whereas 1,013 NEMGs were identified as connected with MT function in a previous study (Ali *et al.* 2019). DE analysis on NEMGs showed that the expression of 174 genes was higher in R_2_C than in RC_2_ for the four development stages, while 148 genes exhibited the opposite expression trend (File S2). Among these DEGs, the largest number in the two triploids was in the blastula period (129 up-regulated genes in R_2_C and 120 up-regulated genes in RC_2_). In contrast, the fewest DEGs (16 up-regulated genes in R_2_C and 18 up-regulated genes in RC_2_) were detected in the hatching period (File S2).

### Expression patterns of nuclear genes regulating mitochondrial genes

**Fig. 6.**
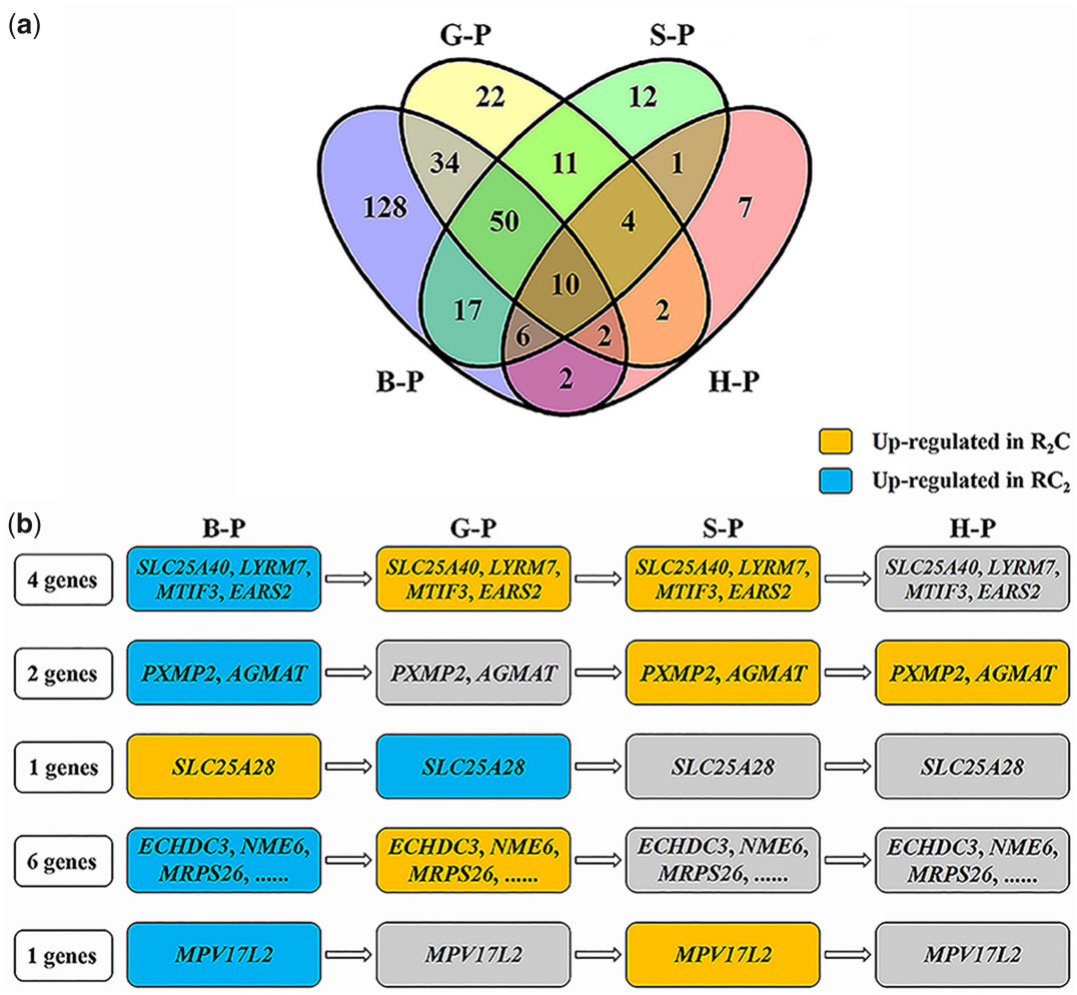
DEGs distribution of the NEMG in the four embryonic development stages. a) Number of DEGs in the four development stages of the triploids. B-P, blastula period; G-P, gastrula period; S-P, segmentation period; H-P, hatching period. b) The expression patterns of 14 genes. Some genes exhibited up-regulated expression in RC_2_ for the first embryonic development stage, while others showed up-regulated expression in R_2_C of the following development stages. The opposite pattern also existed.

To investigate the effects of NEMG expression in embryonic development, the distribution of the DEGs between R_2_C and RC_2_ was detected in the four embryonic development stages. Among the 308 DEGs, 139 DEGs were detected in two or more embryonic development stages between the triploids (Fig. 6a). Among the 139 DEGs, 10 DEGs were found in each of the four development stages. Three of these DEGs were up-regulated in R_2_C (*MRPL40*, *RPS14*, and *AKR7A2*), and 7 were up-regulated in RC_2_ (*NDUFB4*, *MRPL53*, *MDHL*, *VDAC1*, *ATP5F1*, *HIGD2A*, and *IDH1*; Fig. 6a). Interestingly, 14 DEGs showed the opposite trend of DE in different developmental stages (Fig. 6b). Thirteen genes exhibited up-regulated expression in RC_2_ initially but showed up-regulated expression in R_2_C of the following embryonic development stages. The opposite pattern was only detected in *SLC25A28*. Overall, the five patterns of NEMG expression were detected during the four embryonic development stages (Fig. 6b). These results reflected a series of expression changes in NU genes and showed their feedback effect on MT function.

## Discussion

Systematic analysis of multiple developmental stages between the two triploid fishes (R_2_C and RC_2_) provides an effective tool for the study of mitochondrial-nuclear correlations in hybrid systems. From fertilized egg to hatching period, RC_2_ spends more time in embryonic development than R_2_C (Table 1). Meanwhile, faster growth ratios were detected in RC_2_ as compared to RC_2_ (Ren *et al.* 2019). These results shed us insight into how distinct MT genomes and the correlation of MT and NU genes regulate these phenotypic variations.

Changes in MT gene expression always lead to changes in growth and may cause diseases (Pagliarini *et al.* 2008). Moreover, the disruption of the MT transcriptional system was detected in F_2_ hybrids of the marine copepod and was correlated with fitness (Ellison and Burton 2006, 2008). Our results also showed that the DE of NU genes occurred between the two triploids (Figs. 4 and 5). The stable regulation patterns from MT to NU gene expression across the four embryonic development stages help us obtain the 270 NU genes, for which MT genes from MT genetics regulate NU gene expression (Fig. 4). DE between the two triploids was observed in *APC16*, which played a key role in the mitotic divisions of early embryos (Shakes *et al.* 2011). Meanwhile, DE in *Trim33* is necessary for the development of the precardiogenic mesoderm (Rajderkar *et al.* 2019). We inferred that *APC16* and *Trim33* may have potential effects on regulating cell cycle time and embryonic development.

Future research into the correlation of the MT and NU genes will be required to improve this study. These results on MT-NU correlations give us insight into their potential effects on embryonic development time and growth heterosis in hybrids.

## Data availability

All short-read RNA-seq data have been deposited in the NGDC database (National Genomics Data Center) under the following BioProject number: PRJCA003625 (https://ngdc.cncb.ac.cn/bioproject/browse/PRJCA003625). The assembled genome of *C. auratus* was downloaded from Genome Warehouse in BIG Data Center (BioProject number: PRJCA001234) and the assembled genome of *C. carpio* was downloaded from NCBI BioProject database (accession number: PRJNA510861). The MT genomes of *C. auratus* and *C. carpio* were downloaded from accession numbers of AY714387.1 and KF856965.1 in NCBI Nucleotide Database, respectively.

Supplemental material is available at *G3* online.

## Funding

This work was supported by the National Natural Science Foundation of China (31702334, 31730098, U19A2040, 31430088, and 91631305), Natural Science Foundation of Hunan Province Grants (2020JJ5355 and 2022JJ10035), the Key Research and Development Program of Hunan Province (2018NK2072 and 2017NK1031), the Earmarked Fund for China Agriculture Research System (CARS-45), 111 Project (D20007), High-Level Talent Agglomeration Program of Hunan, China (2019RS1044), the Cooperative Innovation Center of Engineering and New Products for Developmental Biology of Hunan Province (20134486), Laboratory of Lingnan Modern Agriculture Project (NT2021008), and Huxiang Young Talent Project of China (Grant No. 2021RC3093).

## Conflicts of interests

The authors declare that they have no competing interests.

## Supplementary Material

jkac197_Supplementary_Data_File_S1

jkac197_Supplementary_Data_File_S2
