# Supplementary material for: Correlations of expression of nuclear and mitochondrial genes in triploid fish
Source: G3 (Bethesda). 2022 Aug 4;12(9):jkac197. doi: 10.1093/g3journal/jkac197 (PMC9434317; doi:10.1093/g3journal/jkac197)
Supplement: jkac197_Supplementary_Data_File_S1 [file jkac197_supplementary_data_file_s1.doc]

**Supplementary information**


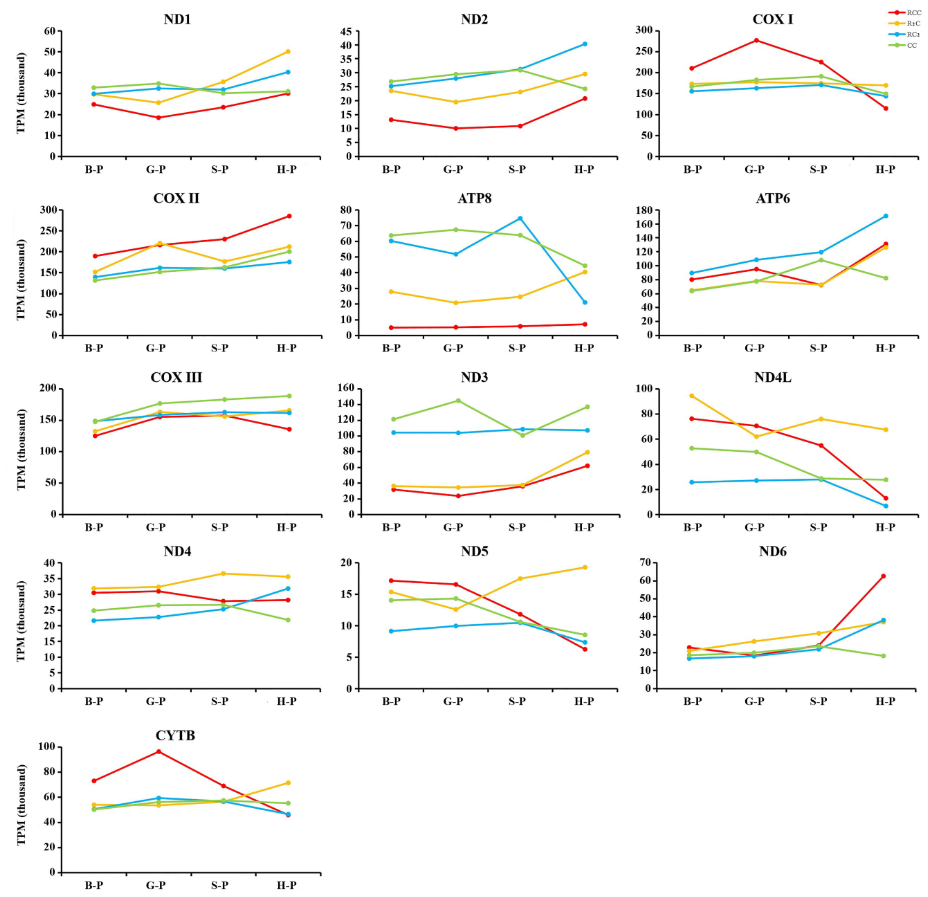


**Fig. S1.** Changing trends in the expression of 13 MT genes during embryonic development. The red line represents RCC; the yellow line represents R_2_C; the blue line represents RC_2_; and the green line represents CC. B-P: blastula period; G-P: gastrula period; S-P: segmentation period; H-P: hatching period.


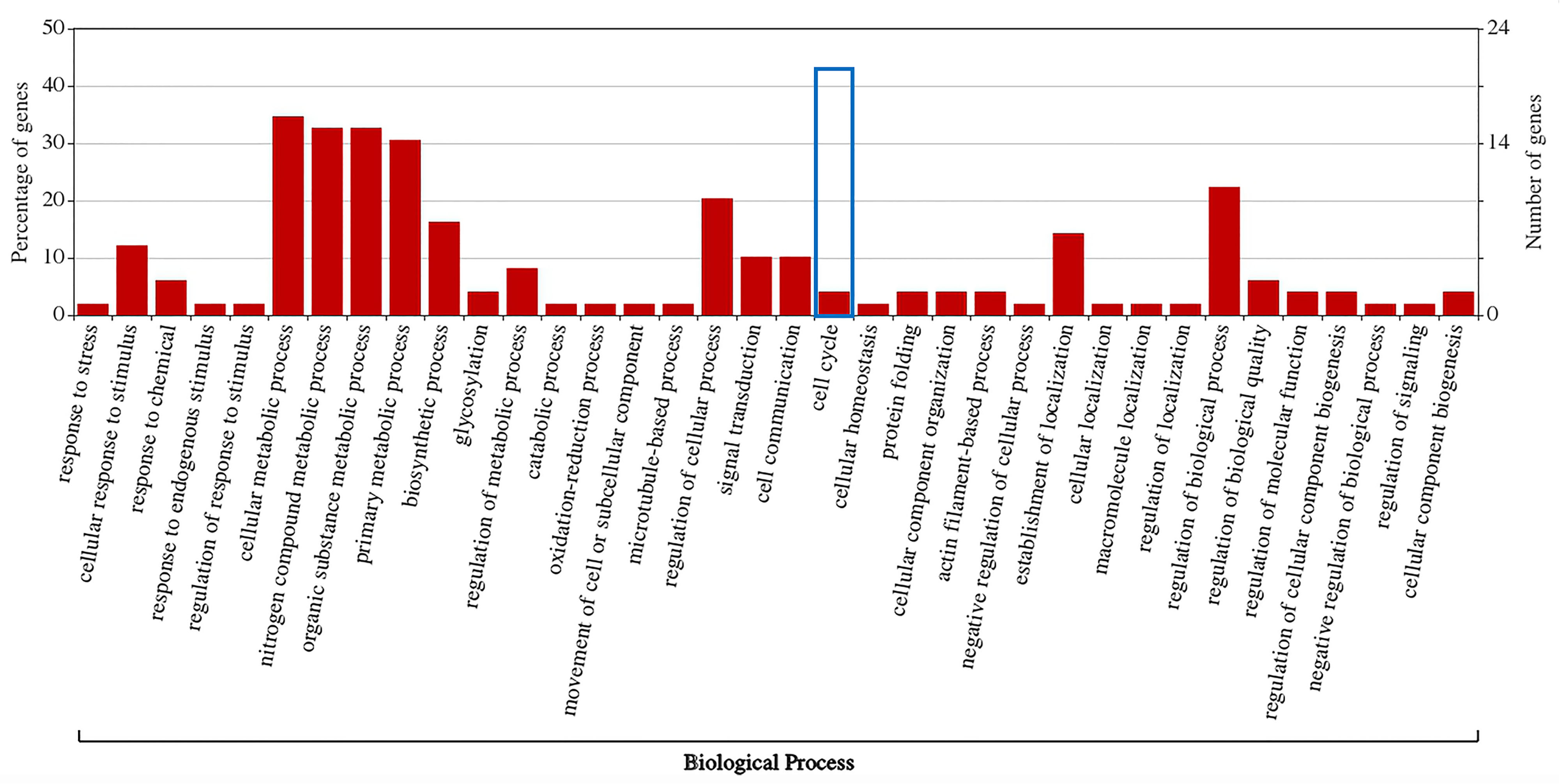


**Fig. S2.** GO analysis of the shared NU genes among the four patterns. Blue box represents the cell cycle related genes (*TRIM33* and *APC16*) were up regulated in RC_2_ than in R_2_C.

**Table S1. Summary of transcriptome data**

| Sample | Clean Reads | Raw Base(G) | Clean Base(G) | Error Rate(%) | Q20(%) | Q30(%) |
| --- | --- | --- | --- | --- | --- | --- |
| CC-B-1 | 32,495,652 | 9.98 | 9.75 | 0.03 | 97.85 | 94.23 |
| CC-B-2 | 26,483,123 | 8.27 | 7.94 | 0.03 | 97.69 | 93.88 |
| CC-B-3 | 24,218,858 | 7.46 | 7.27 | 0.03 | 97.54 | 93.57 |
| CC-G-1 | 34,587,075 | 10.77 | 10.38 | 0.03 | 97.78 | 94.11 |
| CC-G-2 | 27,562,758 | 8.55 | 8.27 | 0.03 | 97.67 | 93.87 |
| CC-G-3 | 33,404,311 | 10.38 | 10.02 | 0.03 | 97.7 | 93.91 |
| CC-S-1 | 23,241,721 | 7.18 | 6.97 | 0.03 | 97.4 | 93.34 |
| CC-S-2 | 28,403,922 | 8.82 | 8.52 | 0.03 | 97.36 | 93.23 |
| CC-S-3 | 28,496,656 | 8.88 | 8.55 | 0.03 | 97.44 | 93.44 |
| CC-H-1 | 32,560,591 | 10.03 | 9.77 | 0.03 | 97.75 | 93.96 |
| CC-H-2 | 26,750,235 | 8.22 | 8.03 | 0.03 | 97.87 | 94.25 |
| CC-H-3 | 25,436,148 | 7.81 | 7.63 | 0.03 | 97.5 | 93.38 |
| RCC-B-1 | 27,643,652 | 8.34 | 8.29 | 0.03 | 97.72 | 93.81 |
| RCC-B-2 | 29,797,063 | 8.99 | 8.94 | 0.03 | 97.79 | 94 |
| RCC-B-3 | 28,209,427 | 8.51 | 8.46 | 0.03 | 97.79 | 94 |
| RCC-G-1 | 33,785,353 | 10.22 | 10.14 | 0.03 | 97.81 | 93.99 |
| RCC-G-2 | 30,768,782 | 9.29 | 9.23 | 0.03 | 97.85 | 94.12 |
| RCC-G-3 | 27,790,217 | 8.38 | 8.34 | 0.03 | 97.68 | 93.72 |
| RCC-S-1 | 25,928,867 | 7.83 | 7.78 | 0.03 | 97.37 | 93.28 |
| RCC-S-2 | 26,807,041 | 8.11 | 8.04 | 0.03 | 97.31 | 93.02 |
| RCC-S-3 | 34,240,991 | 10.44 | 10.27 | 0.03 | 97.53 | 93.66 |
| RCC-H-1 | 24,548,317 | 7.84 | 7.36 | 0.03 | 96.65 | 92.15 |
| RCC-H-2 | 31,122,770 | 9.69 | 9.34 | 0.03 | 96.98 | 92.78 |
| RCC-H-3 | 25,148,519 | 7.94 | 7.54 | 0.03 | 97.17 | 93.08 |
| R_2_C-B-1 | 27,229,150 | 8.36 | 8.17 | 0.03 | 97.83 | 94.16 |
| R_2_C-B-2 | 26,687,786 | 8.2 | 8.01 | 0.03 | 97.82 | 94.13 |
| R_2_C-B-3 | 24,845,720 | 7.64 | 7.45 | 0.03 | 97.67 | 93.81 |
| R_2_C-G-1 | 26,618,790 | 8.21 | 7.99 | 0.03 | 97.59 | 93.69 |
| R_2_C-G-2 | 32,166,864 | 9.99 | 9.65 | 0.03 | 97.4 | 93.34 |
| R_2_C-G-3 | 34,582,921 | 10.71 | 10.37 | 0.03 | 97.31 | 93.15 |
| R_2_C-S-1 | 23,137,078 | 7.11 | 6.94 | 0.03 | 97.49 | 93.5 |
| R_2_C-S-2 | 26,301,506 | 8.16 | 7.89 | 0.03 | 96.84 | 92.09 |
| R_2_C-S-3 | 35,310,900 | 11.01 | 10.59 | 0.03 | 97.72 | 93.91 |
| R_2_C-H-1 | 23,950,424 | 7.51 | 7.19 | 0.03 | 97.55 | 93.59 |
| R_2_C-H-2 | 28,660,816 | 8.81 | 8.6 | 0.03 | 97.26 | 92.97 |
| R_2_C-H-3 | 25,360,903 | 7.92 | 7.61 | 0.03 | 97.47 | 93.41 |
| RC_2_-B-1 | 27,970,905 | 8.76 | 8.39 | 0.03 | 97.17 | 93.07 |
| RC_2_-B-2 | 30,437,254 | 9.49 | 9.13 | 0.03 | 97.19 | 93.09 |
| RC_2_-B-3 | 28,311,037 | 8.92 | 8.49 | 0.03 | 96.99 | 92.75 |
| RC_2_-G-1 | 33,298,930 | 10.31 | 9.99 | 0.03 | 97.09 | 92.72 |
| RC_2_-G-2 | 26,512,432 | 8.23 | 7.95 | 0.03 | 97.16 | 92.98 |
| RC_2_-G-3 | 41,842,884 | 13.03 | 12.55 | 0.03 | 97.18 | 93 |
| RC_2_-S-1 | 27,943,852 | 8.61 | 8.38 | 0.03 | 97.55 | 93.66 |
| RC_2_-S-2 | 28,825,698 | 8.89 | 8.65 | 0.03 | 97.43 | 93.45 |
| RC_2_-S-3 | 29,759,166 | 9.46 | 8.93 | 0.03 | 97.25 | 93.08 |
| RC_2_-H-1 | 29,832,247 | 9.18 | 8.95 | 0.03 | 97.41 | 93.37 |
| RC_2_-H-2 | 32,334,050 | 9.91 | 9.7 | 0.03 | 97.43 | 93.4 |
| RC_2_-H-3 | 29,463,975 | 9.03 | 8.84 | 0.03 | 97.33 | 93.15 |

B: blastula period, G: gastrula period, S: segmentation period, H: hatching period

**Table S2.** Number of maternal mitochondrial reads detected in the two triploids based on transcriptome data

|  | Sample | Blastula | Gastrula | Segmentation | Hatching |
| --- | --- | --- | --- | --- | --- |
| R_2_C | Sample 1 | 98,162(99.98%) | 81,139(99.85%) | 137,212(99.97%) | 344,707(>99.99%) |
|  | Sample 2 | 97,806 (99.97%) | 80,783(99.80%) | 106,390(99.98%) | 419,496(>99.99%) |
|  | Sample 3 | 83,657(99.97%) | 73,421(99.80%) | 102,527(99.96%) | 451,754(>99.99%) |
| RC_2_ | Sample 1 | 439,762(99.16%) | 2,925,015(99.83%) | 1,462,425(99.85%) | 4,281,642(99.99%) |
|  | Sample 2 | 479,339(99.19%) | 3,204,158(99.88%) | 1,452,431(99.89%) | 3,700,428(99.99%) |
|  | Sample 3 | 434,327(99.13%) | 2,927,614(99.79%) | 1,495,960(99.90%) | 5,805,647(99.99%) |

**Table S3.** The *P*-value of *t*-test in MT genes between R_2_C and RCC

| R_2_C *vs.* RCC | Blastula | Gastrula | Segmentation | Hatching |
| --- | --- | --- | --- | --- |
| *ND1* | 0.3146 | 0.0410 | 0.1511 | 0.0042 |
| *ND2* | 0.0535 | 0.0088 | 0.0502 | 0.0160 |
| *COX I* | 0.2773 | 0.0246 | 0.2561 | 0.0090 |
| *COX II* | 0.2326 | 0.7789 | 0.2663 | 0.0028 |
| *ATP8* | 0.0106 | 0.0013 | 0.0035 | 0.0071 |
| *ATP6* | 0.2423 | 0.1431 | 0.2423 | 0.4015 |
| *COX III* | 0.7083 | 0.5051 | 0.9621 | 0.0230 |
| *ND3* | 0.4951 | 0.0267 | 0.8057 | 0.0289 |
| *ND4L* | 0.2838 | 0.3681 | 0.1575 | 0.0022 |
| *ND4* | 0.7507 | 0.5714 | 0.2398 | 0.0048 |
| *ND5* | 0.4164 | 0.0733 | 0.1499 | 0.0013 |
| *ND6* | 0.6108 | 0.0370 | 0.2836 | 0.0046 |
| *CYTB* | 0.1373 | 0.0165 | 0.3597 | 0.0025 |

**Table S4.** The *P*-value of *t*-test in MT genes between RC_2_ and CC

| RC_2_ *vs.* CC | Blastula | Gastrula | Segmentation | Hatching |
| --- | --- | --- | --- | --- |
| *ND1* | 0.2839 | 0.4705 | 0.3098 | 0.0355 |
| *ND2* | 0.4714 | 0.5948 | 0.8286 | 0.0022 |
| *COX I* | 0.4751 | 0.3391 | 0.1088 | 0.5220 |
| *COX II* | 0.5732 | 0.5669 | 0.7749 | 0.0867 |
| *ATP8* | 0.7046 | 0.0372 | 0.6854 | 0.0056 |
| *ATP6* | 0.0954 | 0.0525 | 0.1353 | 0.0013 |
| *COX III* | 0.9504 | 0.3807 | 0.1088 | 0.1061 |
| *ND3* | 0.1362 | 0.0509 | 0.3420 | 0.0801 |
| *ND4L* | 0.0060 | 0.0435 | 0.8362 | 0.0049 |
| *ND4* | 0.1854 | 0.2153 | 0.2990 | 0.0068 |
| *ND5* | 0.0139 | 0.0621 | 0.7430 | 0.0824 |
| *ND6* | 0.2696 | 0.3579 | 0.2690 | 0.0043 |
| *CYTB* | 0.9378 | 0.5540 | 0.7785 | 0.0944 |

**Additional file 2:** The DEGs of nuclear-encoded mt genes in the four embryonic development stages.
